# Morphological and molecular characterization of *Paratylenchus beltsvillensis* n. sp. (Tylenchida: Paratylenchidae) from the rhizosphere of pine tree (*Pinus virginiana* Mill) in Maryland, USA

**DOI:** 10.21307/jofnem-2021-079

**Published:** 2021-09-07

**Authors:** Mihail R. Kantor, Zafar A. Handoo, Sergei A. Subbotin, Gary R. Bauchan, Joseph D. Mowery

**Affiliations:** 1 Mycology and Nematology Genetic Diversity and Biology Laboratory, USDA, ARS, Northeast Area, Beltsville, MD, 20705; 2 Plant Pest Diagnostic Center, California Department of Food and Agriculture, 3294 Meadowview Road, Sacramento, CA, 95832; 3 Center of Parasitology of A.N. Severtsov Institute of Ecology and Evolution of the Russian Academy of Sciences, Leninskii Prospect 33, Moscow, 117071, Russia; 4 Electron and Confocal Microscopy, USDA, ARS, Northeast Area, Beltsville, MD, 20705

**Keywords:** D2-D3 of 28S rRNA gene, ITSrRNA gene, Description, *Pinus virginiana*, Pin nematode, Morphology, Morphometrics, Phylogeny, Scanning electron microscopy, Virginia pine

## Abstract

The pin nematode, *Paratylechus beltsvillensis* n. sp. collected from rhizosphere soil of a Virginia pine tree (*Pinus virginiana* Mill) growing in Little Paint Branch Park, Beltsville, Prince George’s County, Maryland, USA, is described and illustrated along with light and scanning electron photomicrographs. Females, males, and juveniles of this new species were recovered from soil samples using the sugar centrifugal flotation and Baermann funnel extraction methods. Morphologically, females are short, body length ranging from 245 to 267 μm, stylet from 70 to 75 μm long with anchor shaped knobs, vulva located at 70–73% and small vulval flap, spermatheca large, and ovoid filled with sperms. Lateral field with three incisures, of which the outer two are prominent. Tail slender, having a rounded tail terminus. Males without stylet and have a degenerated pharynx, spicules = 17–20 µm and gubernaculum = 5.0–5.5 µm. Both morphological observations and molecular analysis of ITS and partial 28S ribosomal RNA gene sequences indicated that the specimens collected from the soil at Beltsville Park from rhizosphere soil samples from Virginia pine represents a new pin nematode species.

The genus *Paratylenchus* Micoletzky, 1922 is comprised of nematode that are obligate ectoparasites of plants, widely distributed world-wide and associated with a large variety of plants (Ghaderi et al., 2016; Raski, 1991; Siddiqi, 2000; Van den Berg et al., 2014). The first comprehensive review of the genus was given by Tarjan (1960), who made an improvement of the genus diagnosis as well as synonymization of some species. The genus *Gracilacus* (Raski, 1962) was proposed for *Paratylenchus* species with stylet lengths longer than 48 µm, the excretory pore anterior to nerve ring, and well-developed stylet in juveniles (Raski, 1962). However, Brzeski (1963) suggested a synonymization of *Gracilacus* with *Paratylenchus* because the proposed diagnostic characters were unreliable for defining the genera. Although some authors concurred with the synonymy (Brzeski, 1998; Ghaderi et al., 2016; Siddiqi and Goodey, 1964), others accepted *Gracilacus* as a valid genus (Abdel-Rahman and Maggenti, 1988; Brzeski, 1995; Doucet, 1994; Esser, 1992; Geraert, 1965; Huang and Raski, 1986; Raski, 1991; Shahina and Maqbool, 1993; Van den Berg and Buckley, 1993) or a subgenus of *Paratylenchus* (Siddiqi, 2000). In the book on Tylenchulidae, Ghaderi et al. (2016) recognized 117 species of *Paratylenchus*.

With the advent of molecular biology, phylogenetic studies have been conducted to examine the relationships among paratylenchids. Subbotin et al. (2005) were the first to provide molecular characterization of several *Paratylenchus* species using partial 28S rRNA gene sequences. Lopez et al. (2013) used the ITS1 rRNA gene to reconstruct paratylenchid relationships including *G. bilineata* Brzeski (1995) and *G. aculenta* (Brown, 1959; Raski, 1962). Van Den Berg et al. (2014) inferred phylogenetic relationships among several *Paratylenchus* spp. using 28S rRNA (58 sequences) and ITS rRNA (40 sequences) gene sequences for this genus. Wang et al. (2016a), also using the ITS rRNA gene, demonstrated that their newly described species *P. nanjingensis* which has a 64–68 µm long stylet grouped with *P. bilineatus* and *P. aculentus*. In another study, Wang et al. (2016b) described *P. guangzhouensis*, a species with the stylet averaging 47 µm long and based on their ITS rRNA phylogeny, the authors showed that this species was clustered with those four species having a long stylet. Recently, Munawar et al. (2021), Singh et al. (2021), Clavero-Camacho et al. (2021) published comprehensive phylogenies of the genus *Paratylenchus*. These phylogenetic analyses did not support a justification of erection for the genus *Gracilacus* and this genus was considered as a synonym of *Paratylenchus*.

During a nematological survey, an unknown *Paratylenchus* species with a long stylet was found in a rhizosphere soil of a Virginia pine tree (*Pinus virginiana* Mill) in Beltsville, Prince George’s County, Maryland, USA. Morphological and molecular examination of nematode specimens revealed that they belong to a new species, which named here *Paratylechus beltsvillensis* n. sp. The objective of this study was also to describe this new species using light (LM) and scanning electron microscopy (SEM) and provide its molecular characterization.

## Materials and methods

### Nematode samples

In September and October of 2020, few soil samples were collected in the Little Paint Branch Park, Beltsville, Prince George’s County, Maryland, USA And sent to the Plant Pest Diagnostic Center, California Department of Food and Agriculture, Sacramento, CA and part of the same soil samples were analyzed at the Mycology and Nematology Genetic Diversity and Biology Laboratory USDA, ARS (MNGDBL), Beltsville. Nematodes were recovered from soil samples using the sugar centrifugal flotation and Baermann Funnel extraction methods (Jenkins, 1968).

### Morphological examination

Nematodes were fixed in 3% formaldehyde and processed to glycerin by the formalin glycerin method (Golden, 1990; Hooper, 1970). Photomicrographs of the specimens were taken with a Nikon Eclipse Ni compound microscope using a Nikon DS-Ri2 camera. Specimens were measured with an ocular micrometer on Leitz DMRB compound microscope. Nematodes were observed with the low-temperature scanning electron microscopy (LT-SEM) using the techniques described in Kantor et al. (2020) and Carta et al. (2020).

### DNA extraction, PCR, sequencing, and phylogenetic analysis

DNA was extracted from several specimens using the proteinase K protocol. DNA extraction, PCR, and cloning protocols were as described by Tanha Maafi et al. (2003). The primer sets: D2A (5′ – ACA AGT ACC GTG AGG GAA AGT TG – 3′) and D3B (5′ – TCG GAA GGA ACC AGC TAC TA – 3′) amplifying the D2-D3 of 28S rRNA gene (Subbotin et al., 2006), TW81 (5′ – GTT TCC GTA GGT GAA CCT GC – 3′) and AB28 (5′ – ATA TGC TTA AGT TCA GCG GGT – 3′) amplifying ITS rRNA (Tanha Maafi et al., 2003) were used in this study. PCR products were purified using the QIAquick Gel Extraction Kit (Qiagen) following the manufacturer’s instructions and submitted to direct sequencing at GENEWIZ (USA, CA). The new *Paratylenchus* sequences were submitted to the GenBank database under accession numbers: MW413581, MW413582 (28S rRNA gene), and MW413587 (ITS rRNA gene).

The new sequences for each gene (D2–D3 of 28S rRNA, ITS rRNA) were aligned using ClustalX 1.83 (Thompson et al., 1997) with previous published DNA sequences (Etongwe et al., 2020; Munawar et al., 2018; Mwamula et al., 2020; Singh et al., 2021; Subbotin et al., 2005, 2006; Van den Berg et al., 2014; Wang et al., 2016a, b). ClustalX with the modified parameters (gap opening = 5 and gap extension = 3) were applied to generate the D2–D3 of 28S rRNA and ITS rRNA gene alignments. Sequence datasets were analyzed with Bayesian inference (BI) (Ronquist and Huelsenbeck, 2003) using MrBayes 3.1.2 as described by Van den Berg et al. (2014).

## Results and discussion

*Paratylenchus beltsvillensis* n. sp.

(Figs. 1–3)

**Figure 1:**
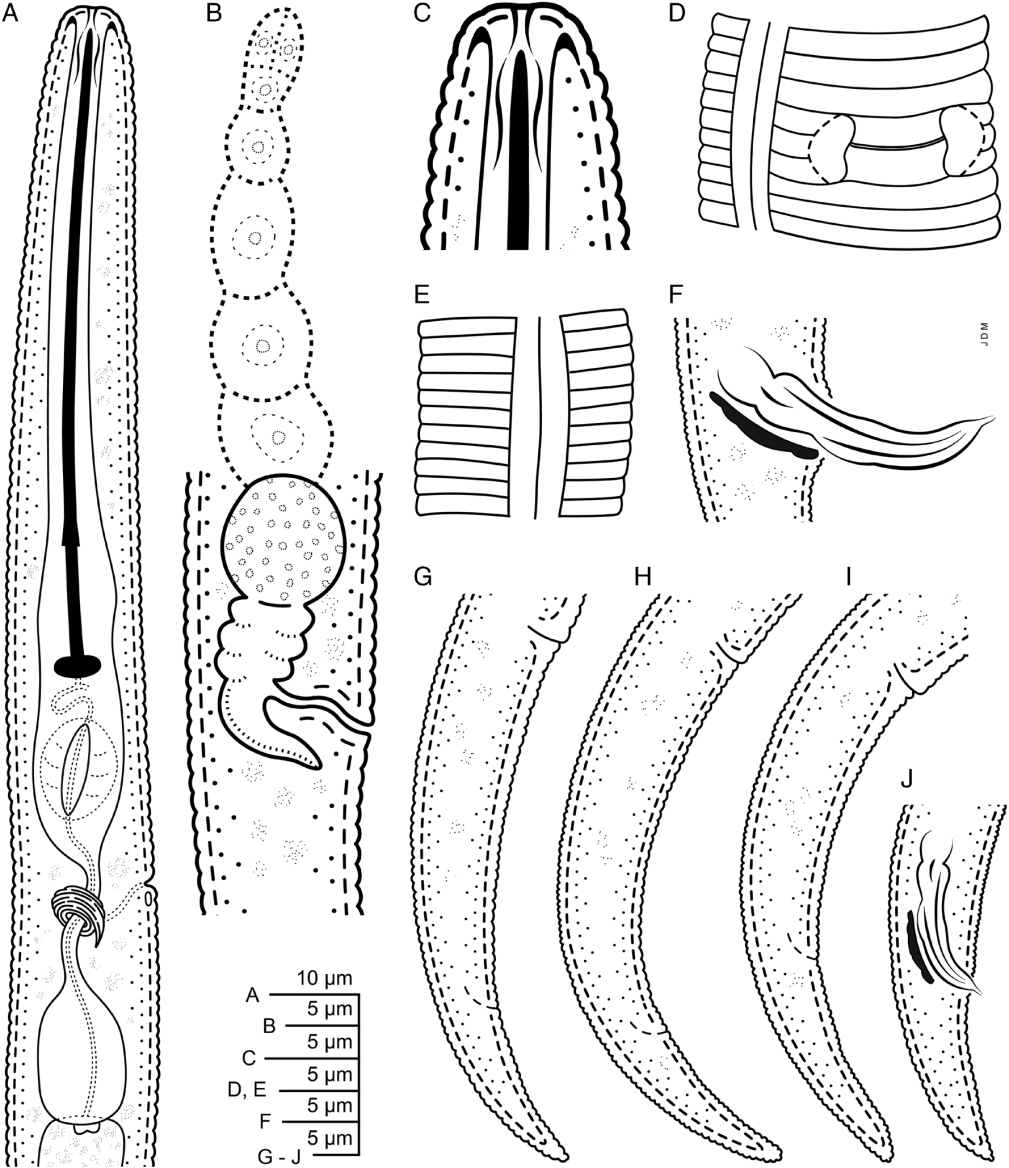
Line drawings of *Paratylenchus beltsvillensis* n. sp. A: Female pharyngeal region; B: Vulval region with vulva, uterus, and spermatheca; C: Female lip region with stylet; D: Female specimen with vulval opening; E: Lateral field (mid-body); F: Male specimen with spicules and gubernaculum; G–I: Female tails with vulval opening and tail variations; J: Male tale showing spicules and gubernaculum.

**Figure 2:**
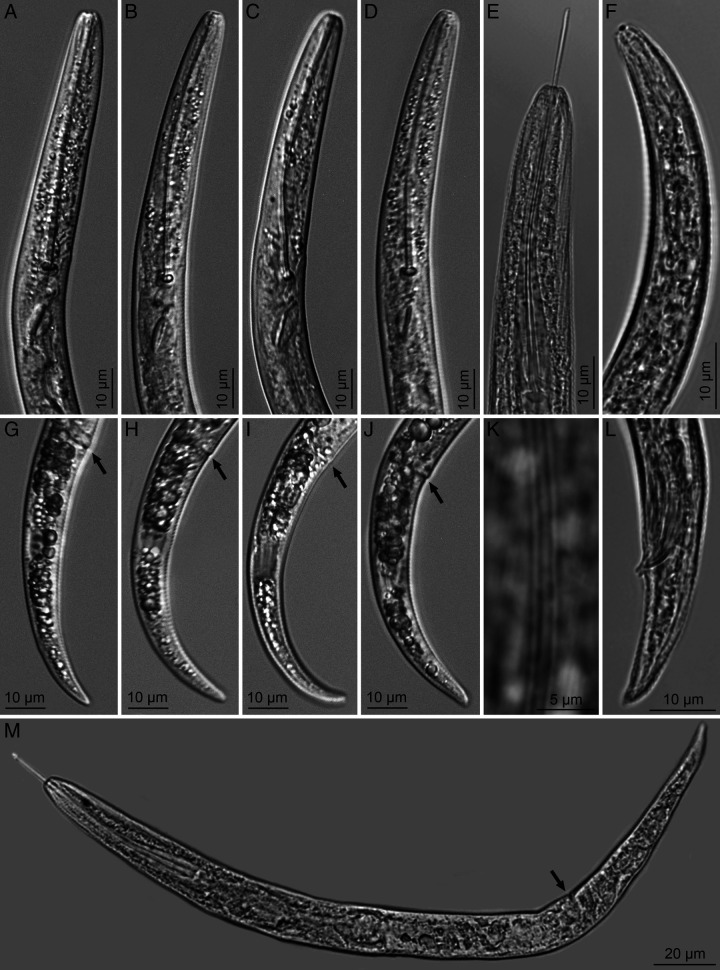
Photomicrographs of *Paratylenchus beltsvillensis* n. sp. A–E: Female anterior ends; F: Male anterior end; G–J: Female posterior ends with vulva area (arrows) and tails; K: Lateral field (mid-body); L: Male posterior end with spicules; M: Whole female specimen (arrow pointing to vulva).

**Figure 3:**
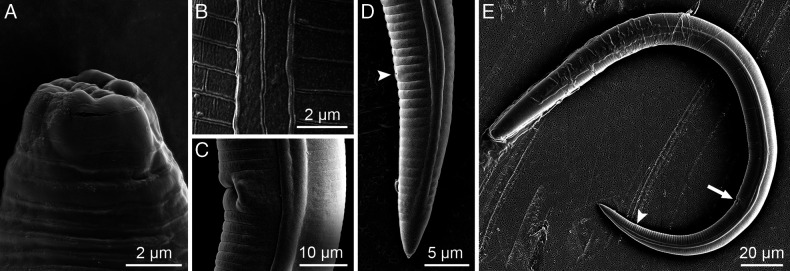
SEM images of *Paratylenchus beltsvillensis* n. sp. A: Female specimen, head; B: Lateral field (mid-body); C: Female specimen, arrow showing the vulva opening; D: Female posterior end, arrow showing the anal opening; E: Whole female specimen with arrows showing vulval and anal openings.

Measurements: See Table 1.

**Table 1.** Morphometrics of *Paratylenchus beltsvillensis* n. sp.

Character	Holotype	Female	Male
n	1	10	3
L	257.0	252.2 ± 8.18 (245.0–267.0)	275.0 ± 5.5 (270.0–280.0)
a	20	18.43 ± 0.6 (18.0–19.0)	11.0 ± 0.02 (10.98–11.02)
b	2.25	2.12 ± 0.07 (1.99–2.23)	3.2 ± 0.1 (3.1–3.2)
c	12.85	11.06 ± 1.63 (9.92–13.61)	14.43 ± 1.25 (13.0–15.3)
Max. body diam.	13.0	13.70 ± 0.67 (13.0–15.0)	25.0 ± 0.5 (24.5–25.5)
Stylet length	70.0	72.25 ± 2.49 (70.0–75.0)	–
Ant end to Ex. Pore	85.0	95.10 ± 4.72 (90.0–100.0)	–
Head to gland tip	114.0	118.80 ± 4.10 (115.0–127.0)	85.0 ± 5.0 (80.0–90.0)
Tail length	20.0	23.19 ± 3.09 (17.0–27.5)	–
*V*%	71.0	71.85 ± 1.07 (70.4–73.4)	–
Spicules	–	–	18.3 ± 1.53 (17.0–20.0)
Gubernaculum	–	–	5.17 ± 0.3 (5.0–5.5)

**Note:** All measurements are in μm and in the form: mean ± standard deviation (s.d.) (range).

### Description

#### Females

Body slender, vermiform, assuming arcuate C-shaped form when killed by gentle heat and tapering uniformly to finely rounded tail tip. Cuticle with transverse striae. Lateral field usually with two lines at curvature near mid body, occasionally an additional faint third line observed between the two outer lines, which were observed under SEM. Lip region flat truncate, continuous with the body contour and bearing 2–3 fine annuli. Cephalic framework weak. Stylet long and slender, flexible. With anchor-shaped knobs. Excretory pore located at the metacorpus level or slightly posterior to it. Hemizonid located 1–2 annuli anterior to excretory pore. Procorpus expanding uniformly into median bulb. Small lateral vulval flapsare visible under SEM. Spermatheca oblong to oval elongate with round spermatozoa. Anus usually indistinct. Tail conical, tapering uniformly to a bluntly rounded tail terminus.

#### Male

Common. Similar to females, except for having stylet and pharynx degenerate and or indistinct. Faint traces of pharynx in a couple of specimens. Spicules cephalate, slightly curved ventrally. Lateral fields with 2 or 3 incisures. Tail elongate conoid, with a bluntly rounded terminus.

### Type host and locality

Associated with roots and soil of Virginia pine (*Pinus virginiana* Mill) trees in the Little Paint Branch Park, Beltsville, Prince George’s County, Maryland, USA. The global positioning coordinates: 39.036071 N, 76.391826 W.

### Type material

Holotype (1 female): Slide T-743t and T-744t (one male), deposited in the United States Department of Agriculture Nematode Collection, Beltsville, MD, USA. Paratypes (Females, and Males): Same data and repository as holotype. Slides T-7463p–T-7470p. Additional females on slide numbers T-7471-p–T-7472p at Plant Pest Diagnostic Center, California Department of Food and Agriculture, Sacramento, CA, USA; T-7473p to T-7474 pat University of California, Riverside, CA, USA; T-7475p to T-7476p, University of California, Davis, CA, USA and T-7477p-T-7478p at Fera, Plant Pest Disease Cultures and Collections, York, United Kingdom.

### Diagnosis and relationships

*Paratylenchus beltsvillensis* n. sp. is characterized by a combination of the following morphological features in females: slender vermiform body (0.24–0.67) mm, long stylet (70–75 µm) with anchor shaped knobs, excretory pore located at the metacorpus level or slightly posterior to it (at 90–100 µm from anterior end), and vulva located at 70–73% with small vulval flap; tail conical, tapering uniformly to a bluntly rounded tail terminus; males have stylet and pharynx degenerate, spicules measuring 17–20 µm and gubernaculum 5.0–5.5 µm.

*Paratylenchus beltsvillensis* n. sp. is similar with *P. nanjingensis* and *P. aculentus*, from which it differs by stylet length (70.0–75.0 vs 64–68 and 48–70 µm) and position of excretory pore (90.0–100.0 vs 55.5–64.5 and 59–89 µm).

The new species is similar to *P. peperpotti* (Schoemaker, 1963), although it differs from the latter by having a slightly smaller *b* value (1.99–2.2 vs 2.0–3.9) and a slightly posterior location of excretory pore (90.0–100.0 vs 61–87 µm).

It differs from *P. aciculus* (Brown, 1959) by having a slightly longer stylet (70–75 vs 61–69 µm), a slightly shorter body length (245–267 vs 240–310 µm), and by the males having a longer spicules (17–20 vs 14.5–16.5 µm).

Also, *P. beltsvillensis* n. sp. it is close to *P. solvaga* (Raski, 1976) and *P. marylandicus* Jenkins, 1960. From *P. solvaga,* it differs by its tail shape, which is mostly deformed, and location of excretory pore. From *P. marylandicus* it differs mostly by shorter body length, number of lateral lines (2–3 vs 4), tail shape.

It also comes close to *P. steneri* Golden, 1961 from which it differs by the number of lateral lines (2–3 vs. 4), longer stylet length (70–75 vs. 65–69 µm), and males present vs absent in *P. steineri*.

### Etymology

The species name is derived from Beltsville, the type locality of this species.

### Molecular analysis

#### The D2–D3 of 28S rRNA gene

**Figure 4:**
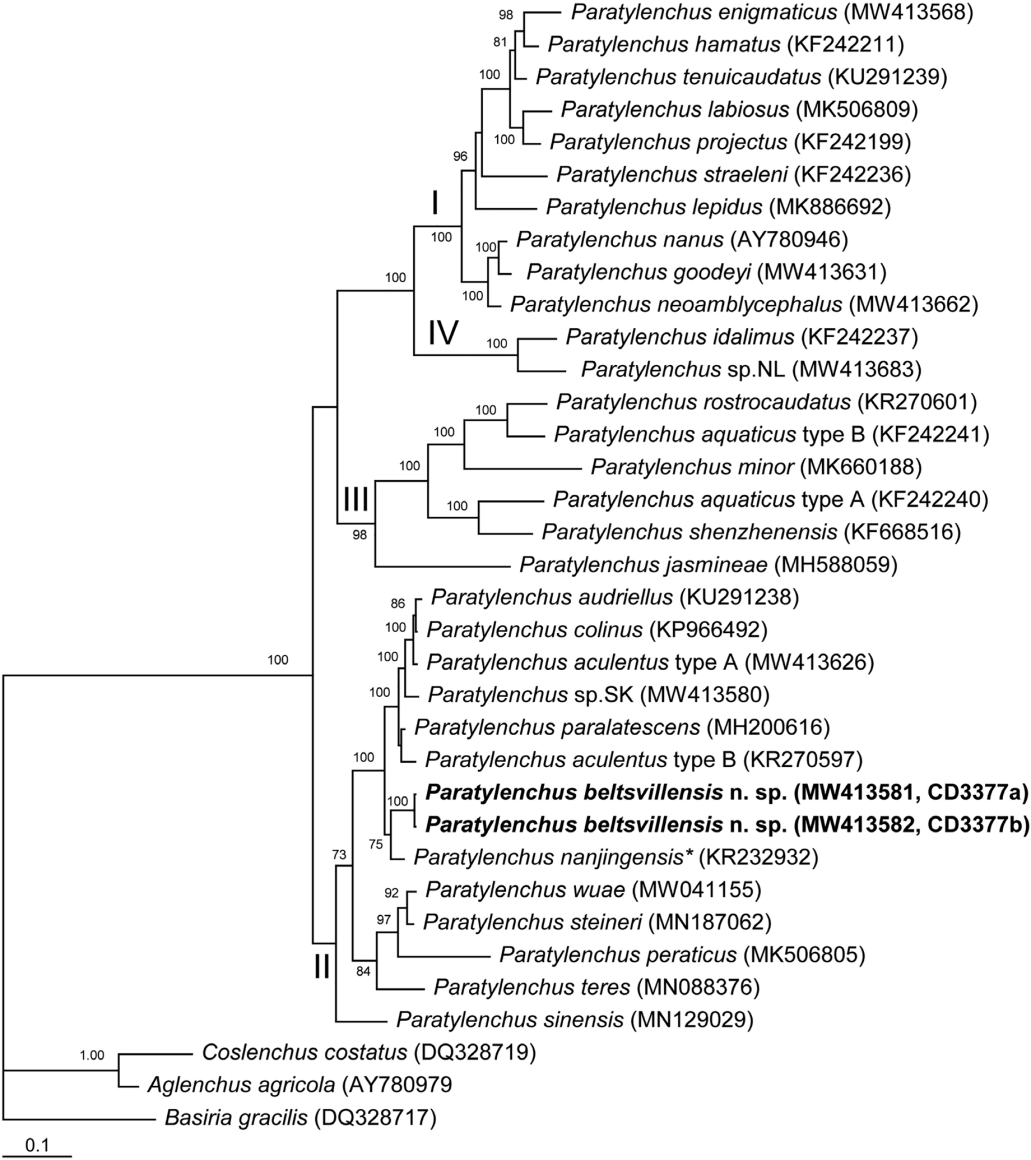
Phylogenetic relationships of *Paratylenchus beltsvillensis* n. sp. with other related species. Bayesian 50% majority rule consensus tree from two runs as inferred from analysis of the D2-D3 of 28S rRNA gene sequence alignment under the GTR + I + G model. Posterior probabilities equal or more than 70% are given for appropriate clades. New sequences are indicated in bold. Clade numbers are given according to Singh et al. (2021). *identified as *Paratylenchus* sp. in the GenBank.

The alignment generated with modified parameters was 782 bp in length and contained 36 sequences, including two sequences of new species and three sequences of outgroup taxa. Phylogenetic relationships of *P. beltsvillensis* n. sp. within selected *Paratylenchus* are given in Figure 4. Sequences of *P. beltsvillensis* n. sp. formed a clade with that of *P. nanjingensis*. (KR232932) collected from soil associated with *Pinus massoniana* in Nanjing, China (Maria et al., unpublished) and differed from this sequence in 27 bp (3.9%).

#### The ITS rRNA gene

**Figure 5:**
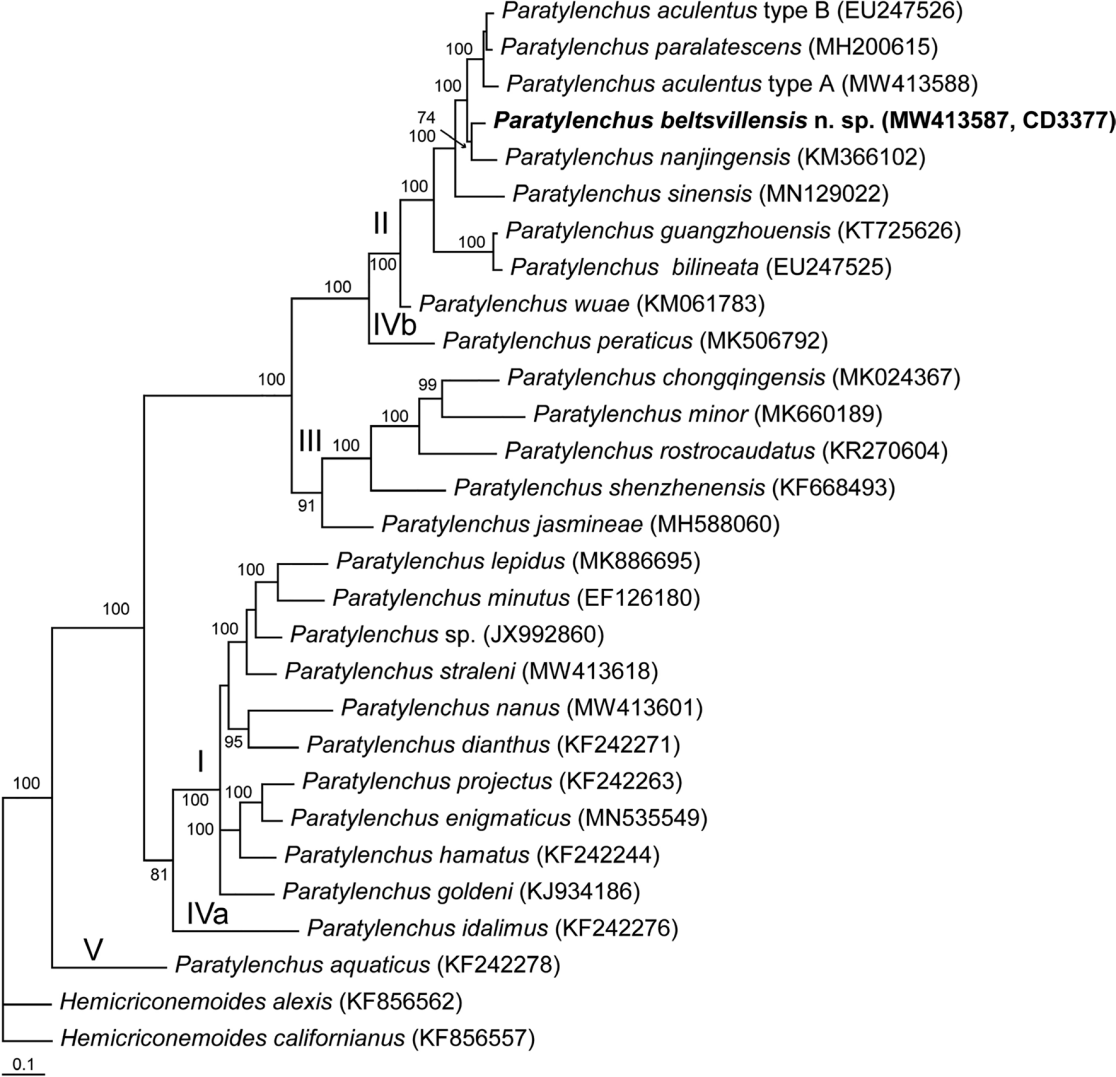
Phylogenetic relationships of *Paratylenchus beltsvillensis* n. sp. with other related species: Bayesian 50% majority rule consensus tree from two runs as inferred from analysis of the ITS rRNA gene sequence alignment under the GTR + I + G model. Posterior probabilities equal or more than 70% are given for appropriate clades. New sequences are indicated in bold. Clade numbers are given according to Singh et al. (2021).

The alignment generated with modified parameters was 1,021 bp in length and contained 21 sequences, including for two outgroups. Phylogenetic relationships of *P. beltsvillensis* n. sp. within selected *Paratylenchus* are given in Figure 5. The sequence of *P. beltsvillensis* n. sp. was inferred to form a clade with that of *P. nanjingensis* and was different from sequences of *P. nanjingensis, P. paralatescens*, and *P. aculentus* type A and *P. aculentus* type B in 47 bp (6.8%), 51 bp (7.4%) 60 bp (8.7%), and 50 bp (7.2%), respectively.

The phylogenetic relationships within *Paratylenchus* species obtained in this study are congruent with those recently presented by Zhuo et al. (2018), Munawar et al. (2021), Singh et al. (2021) and Clavero-Camacho et al. (2021). *Pratylenchus beltsvillensis* n. sp. clustered with species belonging to the Clade II according to Singh et al. (2021). *Paratylenchus beltsvillensis* n. sp. is molecularly close to *P. nanjingensis, P. aculentus, P. audriellus, P. paralatescens*, and *P. colinus.* This clade contains species having stylet longer than 43 µm, two-three incisures in a lateral field and advulval flaps.

In conclusion, both morphological and molecular observations with known and closely related species indicate that *Paratylenchus* species isolated from rhizosphere soil of a Virginia pine tree represents a new pin nematode species, described here as *Paratylenchus beltsvillensis* n. sp.

## References

[R1] Abdel-Rahman, F. and Maggenti, A. R. 1988. *Gracilacus elongata* n. sp. (Nemata: Criconematioidea) parasitic on *Juncus ensifolius* Wikstr. from Mendocino, California. Revue de Nématologie 11:303–306.

[R2] Brown, G. L. 1959. Three new species of the genus *Paratylenchus* from Canada (Nematoda: Criconematidae). Proceedings of the Helminthological Society of Washington 25:1–8.

[R3] Brzeski, M. W. 1963. *Paratylenchus macrodorus* n. sp. (Nematoda: Paratylenchidae), a new plant-parasitic nematode from Poland. Bulletin de I’Académie Polonaise des Sciences. Classe II 11:277–280.

[R4] Brzeski, M. W. 1995. Paratylenchinae: morphology of some known species and descriptions of *Gracilacus bilineata* sp. n. and *G. vera* sp. n. (Nematoda: Tylenchulidae). Nematologica 41:535–565.

[R5] Brzeski, M. W. 1998. Nematodes of Tylenchina in Poland and temperate Europe Warszawa, Poland: Muzeum I Instytut Zoologii PAN, p. 397.

[R6] Carta, L. K., Handoo, Z. A., Li, S., Kantor, M., Bauchan, G., McCann, D., Gabriel, C. K., Yu, Q., Reed, S., Koch, J., Martin, D. and Burke, D. J. 2020. Beech leaf disease symptoms caused by newly recognized nematode subspecies *Litylenchus crenatae mccannii* (Anguinata) described from *Fagus grandifolia* in North America. Forest Pathology 50:e12580.

[R7] Clavero-Camacho, I., Palomares-Rius, J. E., Cantalapiedra-Navarrete, C., Leon-Ropero, G., Martin-Barbarroja, J., Archidona-Yuste, A. and Castillo, P. 2021. Integrative taxonomy reveals hidden cryptic diversity within pin nematodes of the genus *Paratylenchus* (Nematoda: Tylenchulidae). Plants 10:1454. 34371658 10.3390/plants10071454 PMC8309243

[R8] Doucet, M. E. 1994. New data on *Gracilacus colina* Huang & Raski, 1986 (Nemata: Criconematoidea). Fundamental and Applied Nematology 17:117–121.

[R9] Esser, R. P. 1992. A diagnostic compendium to species included in Paratylenchinae Thorne, 1949 and Tylenchocriconematinae Raski & Siddiqi, 1975 (Nematoda: Criconematoidea). Nematologica 38:146–163.

[R10] Etongwe, C. M., Singh, P. R., Bert, W. and Subbotin, S. A. 2020. Molecular characterisation of some plant-parasitic nematodes (Nematoda: Tylenchida) from Belgium. Russian Journal of Nematology 28:1–28.

[R11] Geraert, E. 1965. The genus *Paratylenchus*. Nematologica 11:301–334.

[R12] Ghaderi, R., Geraert, E. and Karegar, A. 2016. The Tylenchulidae of the world. Identification of the family Tylenchulidae (Nematoda: Tylenchida). Academia Press, Gent, Belgium, p. 453.

[R13] Golden, A. M. 1990. “Preparation and mounting nematodes for microscopic observations”, In Zuckerman, B. M., Mai, W. F. and Krusberg, L. R. (Eds), Plant Nematology Laboratory Manual Amherst, MA: University of Massachusetts Agricultural Experiment Station, pp. 197–205.

[R14] Hooper, D. J. 1970. “Handling, fixing, staining, and mounting nematodes”, In Southey, J. F. (Ed.), Laboratory Methods for Work with Plant and Soil Nematodes, 5th ed. London: Her Majesty’s Stationery Office, pp. 39–54.

[R15] Huang, C. S. and Raski, D. J. 1986. Four new species of *Gracilacus* Raski, 1962 (Criconematoidea: Nemata). Revue de Nématologie 9:347–356.

[R16] Jenkins, W. R. 1968. A rapid centrifugal flotation technique for separating nematodes from soil. Plant Disease Reporter 48:692.

[R17] Kantor, M., Handoo, Z. A., Skantar, A. M., Hult, M. N., Ingham, R. E., Wade, N. M., Ye, W., Bauchan, G. R. and Mowery, J. D. 2020. Morphological and molecular characterisation of *Punctodera mulveyi* n. sp. (Nematoda: Punctoderidae) from a golf course green in Oregon, USA, with a key to species of *Punctodera*. Nematology 23:667–683.

[R18] Lopez, M. A. C., Robbins, R. and Szalanski, A. L. 2013. Taxonomic and molecular identification of *Hemicaloosia, Hemicycliophora, Gracilacus* and *Paratylenchus* species (Nematoda: Criconematidae). Journal of Nematology 45:145–171. 24115782 PMC3792835

[R20] Munawar, M., Yevtushenko, D. P., Palomares-Rius, J. E. and Castillo, P. 2021. Species diversity of pin nematodes (*Paratylenchus* spp.) from potato growing regions of Southern Alberta, Canada. Plants 10:188. 33498173 10.3390/plants10020188 PMC7908996

[R19] Munawar, M., Powers, T. O., Tian, Z. L., Harris, T. S., Higgins, R. and Zheng, J. W. 2018. Description and distribution of three criconematid nematodes from Hangzhou, Zhejiang province, China. Journal of Nematology 50:183–206. 10.21307/jofnem-2018-010 PMC6909330 30451437

[R21] Mwamula, O. A., Kabir, F. Md, Lee, G., Choi, I. H., Kim, Y. H., Bae, E. -J. and Lee, D. W. 2020. Morphological characterisation and molecular phylogeny of several species of Criconematina (Nematoda: Tylenchida) associated with turfgrass in Korea, as inferred from ribosomal and mitochondrial DNA. Nematology 22:939–956.

[R22] Raski, D. J. 1962. Paratylenchidae n. fam. with descriptions of five new species of *Gracilacus* n. g. and an emendation of *Cacopaurus* Thorne, 1943, *Paratylenchus* Micoletzky, 1922 and Criconematidae Thorne, 1943. Proceedings of the Helminthological Society of Washington 29:189–207.

[R23] Raski, D. J. 1976. Revision of the genus *Paratylenchus* Micoletzky, 1922 and descriptions of new species. Part III of three parts-*Gracilacus*. Journal of Nematology 8:97–115. 19308207 PMC2620159

[R24] Raski, D. J. 1991. “Tylenchulidae in agricultural soils”, In Nickle, W. R. (Ed.), Manual of Agricultural Nematology New York, NY, Marcel Dekker Inc., pp. 761–794.

[R25] Ronquist, F. and Huelsenbeck, J. P. 2003. MRBAYES 3: Bayesian phylogenetic inference under mixed models. Bioinformatics 19:1572–1574. 12912839 10.1093/bioinformatics/btg180

[R26] Schoemaker, R. L. P. W. 1963. *Gracilacus peperpotti* n. sp. (Nematoda, Paratylenchidae) found in a Surinam coffee plantation soil. Nematologica 9:296–299.

[R27] Shahina, F. and Maqbool, M. A. 1993. *Gracilacus musae* n. sp., (Nematoda: Paratylenchinae) from banana field in Sindh, Pakistan. Pakistan Journal of Nematology 11:1–5.

[R28] Siddiqi, M. R. 2000. Tylenchida: parasites of plants and insects, 2nd ed., CABI Publishing, Wallingford, p. 833.

[R29] Siddiqi, M. R. and Goodey, J. B. 1964. The status of the genera and subfamilies of the Criconematidae (Nematoda); with a comment on the position of *Fergusobia*. Nematologica 9:363–377.

[R30] Singh, P. R., Karssen, G., Couvreur, M., Subbotin, S. A. and Bert, W. 2021. Integrative taxonomy and molecular phylogeny of the plant-parasitic nematode genus *Paratylenchus* (Nematoda: Paratylenchinae): linking species with molecular barcodes. Plants 10:408. 33671787 10.3390/plants10020408 PMC7926417

[R32] Subbotin, S. A., Sturhan, D., Chizhov, V. N., Vovlas, N. and Baldwin, J. G. 2006. Phylogenetic analysis of Tylenchida Thorne, 1949 as inferred from D2 and D3 expansion fragments of the 28S rRNA gene sequences. Nematology 8:455–474.

[R31] Subbotin, S. A., Vovlas, N., Crozzoli, R., Sturhan, D., Lamberti, F., Moens, M. and Baldwin, J. G. 2005. Phylogeny of Criconematina Siddiqi, 1980 (Nematoda: Tylenchida) based on morphology and D2-D3 expansion segments of the 28S rRNA gene sequences with application of a secondary structure model. Nematology 7:927–944.

[R33] Tanha Maafi, Z., Subbotin, S. A. and Moens, M. 2003. Molecular identification of cyst-forming nematodes (Heteroderidae) from Iran and a phylogeny based on the ITS sequences of rDNA. Nematology 5:99–111.

[R34] Tarjan, A. C. 1960. Key to *Paratylenchus* species. Annals of the New York Academy of Sciences 84:338–387.

[R35] Thompson, J. D., Gibson, T. J., Plewniak, F., Jeanmougin, F. and Higgins, D. G. 1997. The CLUSTAL_X windows interface: flexible strategies for multiple sequence alignment aided by quality analysis tools. Nucleic Acids Research 25:4876–4882. 9396791 10.1093/nar/25.24.4876 PMC147148

[R36] Van den Berg, E. and Buckley, N. H. 1993. *Gracilpaurus crenata* (Corbett, 1966) Ganguly & Khan, 1990 from Lesotho and South Africa (Nemata: Paratylenchinae). Phytophylactica 25:75–80.

[R37] Van den Berg, E., Tiedt, L. R. and Subbotin, S. A. 2014. Morphological and molecular characterisation of several *Paratylenchus* Micoletzky, 1922 (Tylenchida: Paratylenchidae) species from South Africa and USA, together with some taxonomic notes. Nematology 16:323–358.

[R38] Wang, K., Xie, H., Li, Y., Wu, W. J. and Xu, C. L. 2016a. Morphology and molecular analysis of *Paratylenchus nanjingensis* n. sp. (Nematoda: Paratylenchinae) from the rhizosphere soil of *Pinus massoniana* in China. Journal of Helminthology 90:166–173. 26821707 10.1017/S0022149X14000819

[R39] Wang, K., Li, Y., Xie, H., Xu, C. L. and Wu, W. J. 2016b. Morphology and molecular analysis of *Paratylenchus guangzhouensis* n. sp. (Nematoda: Paratylenchinae) from the soil associated with *Bambusa multiplex* in China. European Journal of Plant Pathology 145:255–264.

[R40] Zhuo, K., Liu, X. T., Tao, Y., Wang, H. H., Lin, B. and Liao, J. L. 2018. Morphological and molecular characterisation of three species of *Paratylenchus* Micoletzky, 1922 (Tylenchida: Paratylenchidae) from China, with a first description of the male *P. rostrocaudatus*. Nematology 20:837–850.

